# HIV misdiagnosis in sub-Saharan Africa: performance of diagnostic algorithms at six testing sites

**DOI:** 10.7448/IAS.20.1.21419

**Published:** 2017-07-05

**Authors:** Cara S. Kosack, Leslie Shanks, Greet Beelaert, Tumwesigye Benson, Aboubacar Savane, Anne Ng’ang’a, Bita Andre, Jean-Paul BN Zahinda, Katrien Fransen, Anne-Laure Page

**Affiliations:** ^a^ Médecins sans Frontières, Diagnostic Network, Amsterdam, The Netherlands; ^b^ Public Health Department, Médecins sans Frontières, Amsterdam, The Netherlands; ^c^ AIDS Reference Laboratory, Institute of Tropical Medicine, Antwerp, Belgium; ^d^ STD/AIDS Control Program, Ministry of Health Uganda, Kampala, Uganda; ^e^ Laboratoire National de Reference, Conakry, Guinea; ^f^ National AIDS and Sexually Transmitted Infections Control Programme, Nairobi, Kenya; ^g^ Regional Delegation of Public Health for the Littoral Region, Cameroon, Yaoundé; ^h^ Programme National de Lutte contre le Sida et les IST (PNLS), Democratic Republic of Congo, Bukavu; ^i^ Epidemiology and Population Health Department, Epicentre, Paris, France

**Keywords:** HIV, diagnostic, test, RDT, misdiagnosis, algorithm

## Abstract

**Introduction**: We evaluated the diagnostic accuracy of HIV testing algorithms at six programmes in five sub-Saharan African countries.

**Methods**: In this prospective multisite diagnostic evaluation study (Conakry, Guinea; Kitgum, Uganda; Arua, Uganda; Homa Bay, Kenya; Doula, Cameroun and Baraka, Democratic Republic of Congo), samples from clients (greater than equal to five years of age) testing for HIV were collected and compared to a state-of-the-art algorithm from the AIDS reference laboratory at the Institute of Tropical Medicine, Belgium. The reference algorithm consisted of an enzyme-linked immuno-sorbent assay, a line-immunoassay, a single antigen-enzyme immunoassay and a DNA polymerase chain reaction test.

**Results**: Between August 2011 and January 2015, over 14,000 clients were tested for HIV at 6 HIV counselling and testing sites. Of those, 2786 (median age: 30; 38.1% males) were included in the study. Sensitivity of the testing algorithms ranged from 89.5% in Arua to 100% in Douala and Conakry, while specificity ranged from 98.3% in Doula to 100% in Conakry. Overall, 24 (0.9%) clients, and as many as 8 per site (1.7%), were misdiagnosed, with 16 false-positive and 8 false-negative results. Six false-negative specimens were retested with the on-site algorithm on the same sample and were found to be positive. Conversely, 13 false-positive specimens were retested: 8 remained false-positive with the on-site algorithm.

**Conclusions**: The performance of algorithms at several sites failed to meet expectations and thresholds set by the World Health Organization, with unacceptably high rates of false results. Alongside the careful selection of rapid diagnostic tests and the validation of algorithms, strictly observing correct procedures can reduce the risk of false results. In the meantime, to identify false-positive diagnoses at initial testing, patients should be retested upon initiating antiretroviral therapy.

## Introduction

HIV testing algorithms based on rapid diagnostic tests (RDTs) are widely used in HIV testing and counselling (HTC) programmes in areas with limited laboratory infrastructure [1]. RDTs for HIV are low cost, require no cold chain for storage and only minimal training to operate and provide same-day results [2,3]. Algorithms using RDTs are thus ideal for use in resource-constrained settings lacking the laboratory infrastructure and human and financial resources to support the use of more complex techniques, such as enzyme-linked immuno-sorbent assay (ELISA) or immunoblots. To diagnose HIV in these contexts, the World Health Organization (WHO) recommends the sequential use of two or three RDTs in high- and low-prevalence HIV settings, respectively [1]. Unfortunately, these recommendations have yet to be widely implemented: many countries still use serial tiebreaker algorithms wherein two out of three positive RDTs constitute an HIV-positive diagnosis [4].

Furthermore, the WHO recommends using serological assays/RDTs with a sensitivity of at least 99%; the first RDT should have a specificity of at least 98%, while the second and third RDTs should have a specificity of at least 99%. Despite the good performance of numerous individual RDTs in recent WHO evaluations [2,3], false-positive results have been reported from projects operated by Médecins sans Frontières (MSF) [5–7], a humanitarian emergency organization, and by other actors [7–17]. A false-positive result is likely to be psychologically traumatic to the patient and may trigger inappropriate, potentially harmful treatment [6]. Additionally, reporting false-positive results, even if due to a test’s technical limitations, can undermine patient confidence in the HTC centre [6].

We conducted a standardized multicentre study in six sites in sub-Saharan Africa to evaluate the performance of HIV testing algorithms routinely used across the region in real-life conditions. The objectives of this study were to evaluate the performance of the algorithms used in each site and performed in routine conditions; to evaluate the accuracy of the most commonly used RDTs under controlled laboratory conditions and deduce the performance when combined in algorithms following WHO recommendations; and compare these real conditions and ideal performance to WHO-recommended thresholds. Here, we describe the accuracy of the algorithms performed in these sites in routine conditions as compared to a state-of-the-art algorithm of the AIDS reference laboratory at the Institute of Tropical Medicine (ITM), Antwerp, Belgium.

## Methods

### Study settings

This multicentre study took place within six HTC sites in sub-Saharan Africa: (a) Centre Communautaire Matam in Conakry, Guinea; (b) Madi Opei Clinic and Kitgum Matidi Clinic in Kitgum, Uganda; (c) Homa Bay District Hospital in Homa Bay, Kenya; (d) Arua District Hospital in Arua, Uganda; (e) Nylon Hospital in Doula, Cameroun and (f) Baraka Hospital in Baraka, Democratic Republic of Congo (DRC). These HTC sites feature both voluntary and provider-initiated testing. Several sites reported a high positivity rate likely due to the fact that testing focused on spouses of known HIV-positive patients and that, as the main HIV facility in the region, the sites attracted individuals of all risk profiles.

### Study population and inclusion

The study population included all clients greater than equal to five years old attending one of the study HTC sites for HIV testing who provided written informed consent to participate in the study. All participants were invited to sign a separate form for tracing in case of a misdiagnosis.

After enrolment, clients were counselled and tested for HIV according to site-specific procedures and testing algorithms. In addition, blood samples were drawn and EDTA plasma prepared and stored at −20°C for transfer to the reference laboratory.

### Sample size and sampling strategy

We calculated a sample size of at least 200 algorithm HIV-positive and 200 algorithm HIV-negative samples from clients at each study site based on the assumption that both sensitivity and specificity of both sample sets were 98%, providing a 95% confidence interval of ≤±2% for both sensitivity and specificity.

If the prevalence of positive results at the HTC site was between 40% and 60%, we collected all consecutive samples and calculated the total sample size based on prevalence in order to obtain at least 200 HIV-positive and 200 HIV-negative samples (i.e. highest of 200/*p* or 200/(1 − *p*) − maximum sample size 500). This sample size was then increased by 10% to account for losses, problems in shipment, or sample integrity.

If the prevalence was below 40%, we obtained a subset of HIV-positive and HIV-negative samples (according to the algorithm in place). Since the HIV testing algorithms were expected to be relatively accurate, we anticipated very few misdiagnoses. Conservatively, assuming 10% misdiagnosis, we collected a subsample of 220 HIV-positive and 220 HIV-negative samples according to the algorithm. This would ensure that we have at least 200 true-positive and 200 true-negative samples. All samples with inconclusive algorithm results (i.e. discordant with two RDTs in sites not using a tiebreaker) were also collected, along with a backup sample from each participant in case of shipment problems or for potential retesting on site. Every misdiagnosed participant who had consented to tracing was subsequently traced; if the participant had consented, a new sample was collected and tested to exclude the possibility of clerical and other errors.

### Testing strategies and algorithms at study sites

**Figure 1.**
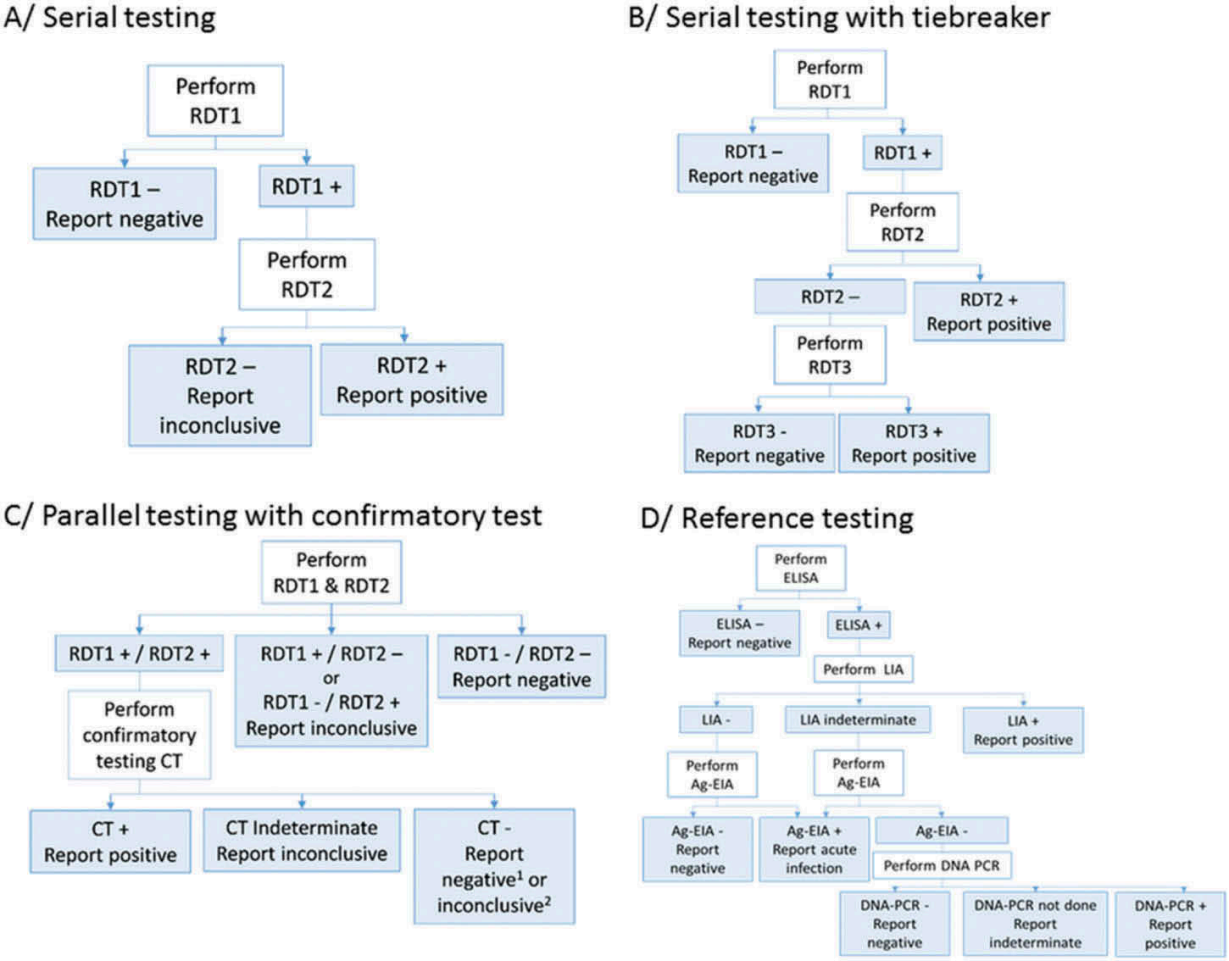
Flow charts of HIV testing strategies and algorithms used. RDT: Rapid diagnostic test; CT: confirmatory test; ELISA: enzyme-linked immunosorbent assay; LIA: line immunoassay; Ag-EIA: antigen-enzyme immunoassay; DNA-PCR: deoxyribonucleic acid–polymerase chain reaction. ^1^In Baraka, DRC; ^2^in Kitgum, Uganda.

Testing strategies – serial versus parallel testing, with and without confirmatory testing – varied among the six study sites (Table 1 and Figure 1). The sample types included capillary whole blood and EDTA plasma. All study sites used Determine HIV-1/2 (Alere, USA) as the first test in the algorithm while at two sites, Baraka and Kitgum, parallel testing with Determine HIV-1/2 and another RDT was performed. The second and third tests used were ImmunoFlow HIV 1–HIV 2 (Core Diagnostics, UK), Uni-Gold HIV (Trinity Biotech, Ireland), HIV 1/2 Stat-Pak (Chembio, USA), ImmunoComb II HIV 1&2 BiSpot (Orgenics, Israel) or GS HIV-1/HIV-2 PLUS O EIA (Bio-Rad, USA), as detailed in Table 1. All tests were performed and interpreted by the staff members who routinely perform testing in the programme, including laboratory technicians and/or counsellors trained on the use of the test. No specific training on test procedures was provided as part of the study so that the results would be representative of routine testing methods.

**Table 1.** HIV testing strategy and algorithms per study site.

	Conakry, Guinea	Kitgum, Uganda	Arua, Uganda	Homa Bay Kenya	Douala, Cameroun	Baraka, DRC	Baraka*, DRC	Kitgum*, Uganda
Testing strategy	Serial	Serial with tie-breaker	Serial with tie-breaker	Serial with tie-breaker	Serial	Serial	Parallel with confirmatory testing	Parallel with confirmatory testing
	Determine HIV-1/2 (Alere, USA)	Determine HIV-1/2 (Alere, USA)	Determine HIV-1/2 (Alere, USA)	Determine HIV-1/2 (Alere, USA)	Determine HIV-1/2 (Alere, USA)	Determine HIV-1/2 (Alere, USA)	Determine HIV-1/2 (Alere, USA)	Determine HIV-1/2 (Alere, USA)
Testing algorithm	ImmunoFlow HIV-1–HIV-2 (Core Diagnostics, UK)	Uni-Gold HIV(Trinity Biotech, Ireland)	HIV-1/2 Stat-Pak (Chembio, USA)	Uni-Gold HIV (Trinity Biotech, Ireland)	ImmunoComb II HIV-1&2 BiSpot (Orgenics/Alere, Israel)	Uni-Gold HIV (Trinity Biotech, Ireland)	Uni-Gold HIV (Trinity Biotech, Ireland)	Uni-Gold HIV (Trinity Biotech, Ireland)
	If discordant: retest in 6 weeks	If discordant: HIV 1/2Stat-Pak (Chembio, USA)	If discordant: Uni-Gold HIV (Trinity Biotech, Ireland)	If discordant: GS HIV-1/HIV-2 PLUS O EIA (Bio-Rad, USA) at CDC, Kisumu, Kenya	If discordant: retest in 6 weeks	If discordant: retest in 6 weeks	On all double positives ImmunoComb II HIV 1&2 CombFirm (Orgenics/Alere, Israel)	On all double positives: ImmunoComb II HIV 1&2 CombFirm (Orgenics/Alere, Israel)
Sample type	EDTA plasma	EDTA plasma	Capillary whole blood	Capillary whole blood	EDTA whole blood	Capillary whole blood	EDTA plasma	EDTA plasma

DRC: Democratic Republic of Congo.

*Algorithm using a simple confirmatory assay (ImmunoComb II HIV 1&2 CombFirm, Orgenics, Israel) with an alternative interpretation by Médecins sans Frontières.

In addition to the on-site testing algorithm, MSF used an alternative algorithm in Kitgum and Baraka that used the ImmunoComb II HIV 1&2 CombFirm (Orgenics, Israel) as a simple confirmatory test following two reactive RDTs. The ImmunoComb is an indirect solid-phase enzyme immunoassay (EIA) containing markers for p24 (gag), p31 (pol) and three env-derived protein spots: gp41, gp120 and gp36. The ImmunoComb was interpreted not as the manufacturer instructs but in accordance with strict criteria proposed in an earlier evaluation [7]. In summary, a reaction of 3–4 spots was interpreted as a positive result, a reaction of 1–2 spots as an indeterminate result and no reaction as a negative result. Gp36 was not considered for the alternative interpretation.

### Reference standard

The reference standard algorithm at the AIDS reference laboratory at ITM, Antwerp, Belgium used an ELISA test (Vironostika® HIV Uni-Form II Ag/Ab, bioMérieux, France) for screening of collected plasma samples [2]. All reactive samples were confirmed by a Line-Immunoassay (LIA, i.e. INNO-LIA HIV I/II Score, Innogenetics NV, Ghent, Belgium) [2,18–20].

The INNO-LIA HIV I/II Score was used to confirm the presence of antibodies against HIV type 1 (HIV-1), including group O viruses, and type 2 (HIV-2). The INNO-LIA HIV I/II Score detects antibodies against gp120, gp41, p31, p24, p17, gp105 and gp36.

If the INNO-LIA HIV I/II Score was negative or indeterminate, the samples were tested with an antigen-enzyme immunoassay (Ag-EIA, i.e. INNOTEST HIV Antigen mAb, Innogenetics NV, Ghent, Belgium) in order to exclude acute infections [2].

If both the LIA and Ag-EIA were negative, the sample was classified as HIV negative. If the LIA was indeterminate and the Ag-EIA negative, the final result was indeterminate. If the LIA was negative or indeterminate and the Ag-EIA was positive (confirmed by neutralization), it was considered a potential seroconversion or acute infection. If the LIA confirmation could not differentiate between HIV-1 and HIV-2 and the outcome was a simple HIV infection, the specimen was tested with an in-house HIV DNA-PCR for HIV-1 and HIV-2 on the DPS. If the DNA-PCR was positive for HIV-1, HIV-2 or for both HIV-1 and HIV-2, the sample was classified as positive for HIV-1, for HIV-2 or for both viruses, respectively.

### Data management and statistical analysis

EpiData 3.1 software (EpiData, Odense, Denmark) was used to perform data entry at all study sites. At ITM, data were collected in an Excel file. The accuracy of data entry at both ITM and the study sites was monitored by a data clerk who double checked all entries. STATA version 13.1 (StataCorp, College Station, TX, USA) was used to perform statistical analysis.

Results of the testing algorithm at each site were compared to those of the reference standard algorithm to calculate sensitivity, specificity, and predictive values. Participants with an inconclusive result either with the on-site algorithm or with the reference algorithm, as well as those diagnosed as having an acute infection with the reference algorithm, were excluded from the performance analyses. At sites where the sampling strategy introduced verification bias (i.e. Douala, Arua, Kitgum; see sampling strategy), correction was carried out using a Bayesian method proposed by Zhou [21].

### Ethics

The study was approved by the MSF Ethics Review Board and the Ethics Committee of the five countries where the study took place. Separate written informed consent was obtained for participation in the study and tracing in case of misdiagnosis.

## Results

Between August 2011 and January 2015, 14,015 clients were tested for HIV at six HTC sites, and 2786 (19.9%) were included in the study (Table 2). The median age was thirty years (IQR: 22–42) and the proportion of males was 38.1% (IQR: 29.6–48.2%). Most study participants who utilized the HTC service were self-referred (58.3%) or were referred by a partner (18.6%). Other testing was provider initiated by the inpatient department (11.9%), outpatient department (1.7%), antenatal care (6.4%) or the tuberculosis clinic (3.0%) within the same health facility (Table 2).

**Table 2.** Demographic and clinical characteristics by study site.

	Conakry, Guinea	Kitgum, Uganda*	Arua, Uganda	Homa Bay Kenya	Douala Cameroun	Baraka, DRC*	Total**
**Study period**	August 2011–October 2012	September 2011–April 2012	July 2012–January 2013	August 2012–February 2013	August 2013–February 2014	October 2012–January 2015	
**Total tested at site**							
Total, *n*	2033	3159	2971	1003	1239	3610	14,015
Positive, *n* (%)	574 (28.2)	332 (10.5)	386 (13.0)	372 (37.1)	396 (32.0)	288 (8.0)	2348 (16.8)
Negative, *n* (%)	1447 (71.2)	2827 (89.5)	2585 (87.0)	617 (61.5)	826 (66.7)	3252 (90.1)	11,554 (82.4)
Inconclusive, *n* (%)	12 (0.6)	0 (0.0)	0 (0.0)	14 (1.4)	17 (1.4)	70 (1.9)	113 (0.8)
**Offered to participate**							
Total, *n*	724	565	750	735	513	559	3846
**Reasons for non-inclusion**							
Offered to participate; total, *n*	724	565	750	735	513	559	3846
Insufficient sample	260	0	22	3	11	0	296
Refusal to participate	8	74	285	232	34	62	695
Excluded due to anaemia	4	0	0	0	0	0	4
Protocol violation	0	49	0	0	0	0	49
Unknown/Other	6	4	0	0	6	0	16
**Included in the study**							
Total, *n*	446	438	443	500	462	497	2786
**Age and gender**							
Median age (IQR)	29 (22–39)	30 (24–39)	29 (23–37)	30 (23–40)	31 (25–41)	30 (23–39)	30 (24–39)
Males, *n* (%)	132 (29.6)	176 (40.3)	213 (48.2)	201 (40.2)	163 (35.3)	177 (35.6)	1062 (38.1)
**Entry mode**							
Volontary testing, *n* (%)	0 (0)	323 (73.90)	443 (100)	459 (91.8)	211 (45.7)	187 (37.8)	1623 (58.3)
Spouse, *n* (%)	238 (53.4)	20 (4.6)	0 (0)	0 (0)	251 (54.3)	10 (2.0)	519 (18.6)
Referred – TB clinic, *n* (%)	57 (12.8)	2 (0.5)	0 (0)	21 (4.2)	0 (0)	3 (0.6)	83 (3.0)
Referred – IPD, *n* (%)	33 (7.4)	0 (0)	0 (0)	0 (0)	0 (0)	297 (59.8)	330 (11.9)
Referred – OPD, *n* (%)	13 (2.9)	33 (7.5)	0 (0)	0 (0)	0 (0)	0 (0)	46 (1.7)
ANC, *n* (%)	105 (23.3)	54 (12.4)	0 (0)	20 (4.0)	0 (0)	0 (0)	179 (6.4)
Other, *n* (%)	0 (0)	5 (1.1)	0 (0)	0 (0)	0 (0)	0 (0)	5 (0.2)

HTC: HIV Testing and Counseling; ANC: Antenatal Care; DRC: Democratic Republic of Congo.

* Not the Local/National/Ministry of Health standard algorithm has been used in this calculation but an algorithm adjusted by MSF according to previous research on diagnostic accuracy of available HIV tests.

**The figures from the algorithm used by MSF in Kitgum and Baraka have been used to calculate the totals.

Across all testing sites, the HIV positivity rate ranged from 8.0% in Baraka to 63.7% in Conakry (Table 2). Of all 2786 specimens tested at the ITM, 1281 were classified as HIV-1 positive, 1 as HIV-2 positive, 25 as HIV-positive (undifferentiated), 2 as acute infections, 3 as inconclusive and 1474 as negative.

After adjustment for the under-representation of negative results in the study design and exclusion of inconclusive results and acute infections, the sensitivity of the testing algorithms ranged from 89.5% in Arua to 100% in Douala and Conakry (Table 3). The specificity ranged from 98.3% in Douala to 100% in Conakry. The positive predictive value (PPV) ranged from 96.4% in Douala to 100% in Conakry. The negative predictive value (NPV) ranged from 98.3% in Arua to 100% in Conakry, Douala and Baraka.

**Table 3.** HIV testing algorithm performance per study site.

	Conakry, Guinea	Kitgum*, Uganda	Kitgum*_’_^#^, Uganda	Arua*, Uganda	Homa Bay, Kenya	Douala*, Cameroun	Baraka*, DRC	Baraka*_’_^#^, DRC
**Included in the study based on HIV status tested at site**								
Total, *n*	446	438	438	443	500	462	497	497
Positive, *n* (%)	222 (49.8)	218 (49.7)	216 (49.6)	212 (47.9)	223 (44.6)	222 (48.1)	226 (45.5)	221 (44.5)
Negative, *n* (%)	220 (49.3)	220 (50.3)	220 (50.2)	231 (52.1)	277 (55.4)	230 (49.8)	219 (44.1)	220 (44.2)
Inconclusive, *n* (%)	4 (0.9)	0 (0)	2 (0.2)	0 (0)	0 (0)	10 (2.2)	52 (10.5)	56 (11.3)
**Results based on reference standard**								
Positive, *n* (%)	222 (49.8)	214 (48.9)	214 (48.9)	212 (47.9)	224 (44.8)	214 (46.3)	221 (44.5)	221 (44.5)
Negative, *n* (%)	224 (50.2)	222 (50.7)	222 (50.7)	230 (51.9)	276 (55.2)	247 (53.5)	275 (55.3)	275 (55.3)
Indeterminate *n* (%)	0 (0.0)	0 (0.0)	0 (0.0)	1 (0.2)	0 (0.0)	1 (0.2)	1 (0.2)	1 (0.2)
Acute infection (%)	0 (0.0)	2 (0.5)	2 (0.5)	0 (0.0)	0 (0.0)	0 (0.0)	0 (0.0)	0 (0.0)
**Diagnostic performance of algorithm at study site**								
Sensitivity % (95% CI)	100 (98.3–100)	96.2 (78.1–99.4)	96.2 (78.2–99.4)	89.5 (76.2–95.8)	98.7 (96.1–99.7)	100 (98.3–100)	100 (98.3–100)	100 (98.3–100)
Specificity % (95% CI)	100 (98.3–100)	99.8 (99.4–99.9)	99.9 (99.6–100)	99.8 (99.3–99.9)	99.3 (97.4–99.9)	98.3 (96.7–99.1)	98.5 (95.7–99.5)	99.9 (99.7–100)
PPV % (95% CI)	100 (98.3–100)	98.2 (95.3–99.5)	99.1 (96.7–99.9)	98.6 (95.9–99.7)	99.1 (96.8–99.9)	96.4 (93.0–98.4)	97.8 (94.9–99.3)	99.5 (97.5–100)
NPV % (95% CI)	100 (98.3–100)	99.1 (96.7–99.9)	99.5 (97.5–100)	98.3 (95.6–99.5)	98.9 (96.9–99.8)	100 (98.4–100)	100 (98.3–100)	100 (98.3–100)
False positive, *n*	0	4	2	3	2	8	5	1
False negative, *n*	0	1	1	4	3	0	0	0

DRC: Democratic Republic of Congo.

*Adjusted results considering verification bias and excluding indeterminate results on-site and seroconverters by the reference algorithm.

^#^Algorithm using a simple confirmatory assay (ImmunoComb II HIV 1&2 CombFirm, Orgenics, Israel) with an alternative interpretation by Médecins sans Frontières.

Overall, 24 (0.9%) study participants were misdiagnosed, ranging from 0 to 8 participants per site (0–1.7%), with 16 false-positive and 8 false-negative results. Six false-negative specimens were retested using a backup sample and were found to be HIV positive (Table 4). Thirteen of 16 false-positive specimens were similarly retested with the backup sample and 10 remained positive (reactive with the 2 first RDTs), while 2 remained positive with only 1 RDT. In addition, in Douala, all eight clients with a false-positive result were traced and a new sample was drawn to exclude clerical error as the cause of misdiagnosis. One client had a negative result, one had an indeterminate result and the remaining six of eight clients maintained a reactive result with both RDTs. All specimens were again found to be negative by the reference algorithm (Table 4).

**Table 4.** Detailed testing results of false-negative and false-positive misdiagnosed participants at site.

Study site	Sample ID	Test result on site	Status on site	ELISA results reference laboratory	LIA results reference laboratory	Status by reference laboratory	Remarks
**False-negative misdiagnosed participants**							
Kitgum	196	Determine: reactive Uni-Gold: non-reactive Stat-Pak: non-reactive	HIV negative*	OD: 1.766 CO: 0.161 OD/CO: 10.97 Result: reactive	sgp120: − gp41: 1+ p31: − p24:1+ p17:2+	HIV positive	No backup sample
Arua	368	Determine: non-reactive	HIV negative	OD: 3.000 CO: 0.179 OD/CO: ≥16.76 Result: reactive	sgp120: 2+ gp41: 3+ p31: 2+ p24: 3+ p17: 3+ sgp105: − gp36: −	HIV-1 positive	Backup sample: Determine: reactive Stat-Pak: reactive Uni-Gold: reactive
Arua	453	Determine: non-reactive	HIV negative	OD: 3.000 CO: 0.186 OD/CO: ≥16.13 Result: reactive	sgp120: 2+ gp41: 4+ p31: − p24: 4+ p17: 4+ sgp105: − gp36: −	HIV-1 positive	Backup sample: Determine: reactive Stat-Pak: reactive Uni-Gold: reactive
Arua	454	Determine: non-reactive	HIV negative	OD: 3.000 CO: 0.186 OD/CO: ≥16.13 Result: reactive	sgp120: 2+ gp41: 4+ p31:3+ p24: 3+ p17: − sgp105: − gp36: −	HIV-1 positive	Backup sample: Determine: reactive Stat-Pak: reactive Uni-Gold: reactive
Arua	611	Determine: non-reactive	HIV negative	OD: 3.000 CO: 0.173 OD/CO: ≥17.34 Result: reactive	sgp120: 2+ gp41: 3+ p31: − p24: 3+ p17: 2+ sgp105: − gp36: −	HIV-1 positive	Backup sample: Determine: reactive Stat-Pak: non-reactive Uni-Gold: reactive
Homa-Bay	394	Determine: non-reactive Uni-Gold: non-reactive	HIV negative	OD: 3.000 CO: 0.162 OD/CO: 18.52 Result: reactive	sgp120: 3+ gp41: 3+ p31: 2+ p24: 3+ p17: 3+ sgp105: − gp36: −	HIV-1 positive	Backup sample: Determine: reactive Uni-Gold: reactive
Homa-Bay	439	Determine: non-reactive Uni-Gold: non-reactive	HIV negative	OD: 3.000 CO: 0.162 OD/CO: 18.52 Result: reactive	spg120: 2+ gp41: 3+ p31: 2+ p24: 4+ p17: 3+ spg105: − gp36: −	HIV-1 positive	Backup sample: Determine: reactive Uni-Gold: reactive
Homa-Bay	469	Determine: non-reactive Uni-Gold: non-reactive	HIV negative	OD: 3.000 CO: 0.162 OD/CO: 18.52 Result: reactive	spg120: 2+ gp41: 3+ p31: − p24: 3+ p17: 3+ spg105: − gp36: −	HIV-1 positive	No backup sample
**False-positive misdiagnosed participants**							
Kitgum	240	Determine: reactive Uni-Gold: reactive ImmunoComb CombFirm: reactive (p24+, p31+, gp120+, gp41+, p31−, gp36−)	HIV positive	OD: 0.06 CO: 0.174 OD/CO: 0.34 Result: non-reactive	–	HIV negative	No backup sample
Kitgum	596	Determine: reactive Uni-Gold: reactive ImmunoComb CombFirm: reactive (p24+, gp120 +, gp41+, p31−, gp36−)	HIV-positive	OD: 0.066 CO: 0.180 OD/CO: 0.37 Result: non-reactive	–	HIV negative	No backup sample
Arua	342	Determine: reactive Stat-Pak: reactive	HIV positive	OD: 0.049 CO: 0.179 OD/CO: 0.27 Result: non-reactive	–	HIV negative	Backup sample: Determine: reactive Stat-Pak: non-reactive Uni-Gold: non-reactive
Arua	529	Determine: reactive Stat-Pak: reactive	HIV positive	OD: 0.057 CO: 0.170 OD/CO: 0.34 Result: non-reactive	–	HIV negative	Backup sample: Determine: reactive Stat-Pak: reactive Uni-Gold: non-reactive
Arua	622	Determine: reactive Stat-Pak: reactive	HIV positive	OD: 0.067 CO: 0.173 OD/CO: 0.39 Result: non-reactive	–	HIV negative	Backup sample: Determine: reactive Stat-Pak: non-reactive Uni-Gold: non-reactive
Homa-Bay	5	Determine: reactive Uni-Gold: non-reactive EIA GS: reactive	HIV positive	OD: 0.084 CO: 0.261 OD/CO: 0.32 Result: non-reactive	–	HIV negative	Backup sample: Determine: reactive Uni-Gold: non-reactive
Homa-Bay	16	Determine: reactive Uni-Gold: reactive	HIV positive	OD: 0.119 CO: 0.261 OD/CO: 0.46 Result: non-reactive	–	HIV negative	Backup sample: Determine: reactive Uni-Gold: reactive The ELISA test at CDC in Kisumu was negative on the same sample. In addition, a western blot (GS HIV-1 Western Blot, No. 32508, Bio-Rad, USA) was carried out at CDC and had an indeterminate result: gp160+, gp120−, p65−, p55/51−, gp41−, p40+, p31+/−, p24−, p18−
Douala	48	Determine: reactive BiSpot: reactive	HIV positive	OD: 0.043 CO: 0.153 OD/CO: 0.28 Result: non-reactive	–	HIV negative	Backup sample same result as initial testing Second sample approx. three months later was collected: Determine: reactive BiSpot: non-reactive ELISA at reference laboratory: non-reactive
Douala	68	Determine: reactive BiSpot: reactive	HIV positive	OD: 0.050 CO: 0.153 OD/CO: 0.33 Result: non-reactive	–	HIV negative	Backup sample same result as initial testing Second sample approx. three months later was collected: Determine: reactive BiSpot: reactive ELISA at reference laboratory: non-reactive
Douala	307	Determine: reactive BiSpot: reactive	HIV positive	OD: 0.053 CO: 0.154 OD/CO: 0.34 Result: non-reactive	–	HIV negative	Backup sample same result as initial testing Second sample approx. two months later was collected: Determine: non-reactive ELISA at reference laboratory: non-reactive
Douala	356	Determine: reactive BiSpot: reactive	HIV positive	OD: 0.058 CO: 0.166 OD/CO: 0.35 Result: non-reactive	–	HIV negative	Backup sample same result as initial testing Second sample approx. three months later was collected: Determine: reactive BiSpot: reactive ELISA at reference laboratory: non-reactive
Douala	381	Determine: reactive BiSpot: reactive	HIV positive	OD: 0.055 CO: 0.166 OD/CO: 0.33 Result: non-reactive	–	HIV negative	Backup sample same result as initial testing. Second sample approx. 6 weeks later was collected: Determine: reactive BiSpot: reactive ELISA at reference laboratory: non-reactive
Douala	411	Determine: reactive BiSpot: reactive	HIV positive	OD: 0.048 CO: 0.154 OD/CO: 0.31 Result: non-reactive	–	HIV negative	Backup sample same result as initial testing Second sample approx. two months later was collected: Determine: reactive BiSpot: reactive ELISA at reference laboratory: non-reactive
Douala	445	Determine: reactive BiSpot: reactive	HIV positive	OD: 3.122 CO: 0.154 OD/CO: 20.27 Result: reactive	sgp120: − gp41: 2+ p31: − p24: − p17: − sgp105: − gp36: − Innotest: non-reactive	HIV negative	Backup sample same result as initial testing Second sample approx. 6 weeks later was collected: Determine: reactive BiSpot: reactive ELISA at reference laboratory: reactive INNO-LIA: sgp120: −, gp41: 2+, p31: −, p24: −, p17: −, sgp105: −, gp36: − Innotest: non-reactive
Douala	464	Determine: reactive BiSpot: reactive	HIV positive	OD: 0.060 CO: 0.154 OD/CO: 0.39 Result: non-reactive	–	HIV negative	Backup sample same result as initial testing Second sample approx. 4 weeks later was collected: Determine: reactive BiSpot: reactive ELISA at reference laboratory: non-reactive
Baraka	479	Determine: reactive Uni-Gold: reactive (both on capillary whole blood and plasma) ImmunoComb CombFirm: reactive (p24+, p31−, gp120+, gp41+, p31−, gp36−)	HIV positive	OD: 0.048 CO: 0.149 OD/CO: 0.32 Result: non-reactive	–	HIV negative	No backup sample

*HIV negative by Ministry of Health algorithm and inconclusive by alternative Médecins sans Frontières algorithm.

CO: Cutoff; OD: optical density; Determine: Determine HIV-1/2 (Alere, USA); Uni-Gold: Uni-Gold HIV (Trinity Biotech, Ireland); Stat-Pak: HIV 1/2 Stat-Pak (Chembio, USA); ImmunoComb CombFirm = ImmunoComb CombFirm II HIV 1&2 CombFirm (Orgenics, Alere, Israel); EIA GS: GS HIV-1/HIV-2 PLUS O EIA (Bio-Rad, USA); BiSpot: ImmunoComb CombFirm II HIV 1&2 BiSpot (Orgenics, Alere, Israel).

Detailed testing results of 99 participants with discordant results between the first two RDTs are described in Table 5. The number of discordant results by site varied from four in Conakry to 54 in Baraka: this high number is explained the fact that all discordant results, considered inconclusive, were included in the study, and by the long duration of the recruitment period. It should be noted that the proportion of inconclusive results among all clients tested in this site during the study period was 1.9%, which was only marginally higher than the proportion of inconclusive results in other sites where discordant results were classified as inconclusive (Table 2). Of these 99 discordant results, the majority (*n* = 91) were negative by the reference standard (Table 4).

**Table 5.** Detailed testing results of discordant rapid test results per study site.

Study site	No. (%) Discordant samples	Sample IDs	Test result on site	HIV status on site	Test results reference laboratory	HIV status by reference laboratory
Conakry	4 (0.9)	151, 153, 198, 327	Determine: reactive ImmunoFlow: non-reactive	Inconclusive	ELISA: non-reactive	HIV negative
Kitgum	10 (2.3)	82, 617	Determine: non-reactive Uni-Gold: reactive Stat-Pak: non-reactive	HIV negative	ELISA: non-reactive	HIV negative
		114, 123	Determine: reactive Uni-Gold: non-reactive Stat-Pak: non-reactive	HIV negative	ELISA: non-reactive	HIV negative
		138, 519, 559	Determine: reactive Uni-Gold: non-reactive Stat-Pak: non-reactive	HIV negative	ELISA: reactive LIA: non-reactive (sgp120: −, gp41: −, p31: −, p24: −, p17: −, gp105: −, gp36: −)	HIV negative
		148	Determine: reactive Uni-Gold: non-reactive Stat-Pak: reactive ImmunoComb Combfirm: positive (p24+, p31−, gp120+, gp41+, gp36−)	HIV positive	ELISA: reactive LIA: reactive (sgp120: 3+, gp41: 3+, p31: 2+, p24: 4+, p17: 3+, sgp105: −, gp36)	HIV-1 positive
		196	Determine: reactive Uni-Gold: non-reactive Stat-Pak: non-reactive	HIV negative	ELISA: reactive LIA: reactive (sgp120: −, gp41: 1+, p31: −, p24: 1+, p17: 2+, sgp105: −, gp36: −)	HIV-1 positive
		205	Determine: reactive Uni-Gold: non-reactive Stat-Pak: reactive ImmunoComb Combfirm: positive (p24+, p31−, gp120+, gp41+, gp36+)	HIV positive	ELISA: reactive LIA: reactive (sgp120: −, gp41: 2+, p31: −, p24: 2+, p17: − sgp105: −, gp36)	HIV-1 positive
Arua	12 (2.7)	251, 344, 354, 387, 402, 431, 440, 441, 465, 489, 495	Determine: reactive Stat-Pak: non-reactive Uni-Gold: non-reactive	HIV negative	ELISA: non-reactive	HIV negative
		620	Determine: reactive Stat-Pak: non-reactive Uni-Gold: reactive	HIV positive	ELISA: reactive LIA: reactive (sgp120: 3+, gp41: 3+, p31: 3+, p24: 2+, p17: 3+, gp105: −, gp36: −)	HIV-1 positive
Homa-Bay	9 (1.8)	5	Determine: reactive Uni-Gold: non-reactive EIA GS: reactive	HIV positive	ELISA: non-reactive	HIV negative
		66, 138, 184, 298, 422	Determine: reactive Uni-Gold: non-reactive EIA GS: non-reactive	HIV negative	ELISA: non-reactive	HIV negative
		170	Determine: reactive Uni-Gold: non-reactive EIA GS: reactive	HIV positive	ELISA: reactive LIA: reactive spg120: 2+, gp41: 3+, p31: 3+, p24: 4+, p17: 3+, spg105: −, gp36: −)	HIV-1 positive
		177	Determine: reactive Uni-Gold: non-reactive EIA GS: reactive	HIV positive	ELISA: reactive LIA: reactive (spg120: 2+, gp41: 2+, p31: 2+, p24: 4+, p17: 3+, spg105: −, gp36: −)	HIV-1 positive
		449	Determine: reactive Uni-Gold: non-reactive EIA GS: reactive	HIV positive	ELISA: reactive LIA: reactive (spg120: −, gp41: 2+, p31: −, p24: 2+, p17: +, spg105: −, gp36: −)	HIV-1 positive
Douala	10 (2.2)	006, 011, 165, 202, 363, 374, 378, 397, 398, 457	Determine: reactive BiSpot: non-reactive	Inconclusive	ELISA: non-reactive	HIV negative
Baraka	54 (10.9)	24, 28, 56, 68, 83, 84, 85, 96, 98, 104, 105, 120, 128, 139, 140, 147, 160, 161, 174, 184, 191, 196, 197, 203, 228, 231, 239, 260, 277, 296, 300, 318, 320, 328, 334, 341, 364, 367, 368, 370, 376, 389, 396, 408, 409, 420, 429, 443, 444, 471, 472, 474	Determine: reactive Uni-Gold: non-reactive	Inconclusive	ELISA: non-reactive	HIV negative
		86	Determine: non-reactive Uni-Gold: reactive	Inconclusive	ELISA: reactive LIA: reactive (sgp120: − gp41: +/−, p31: −, p24: −, p17: −, sgp105: −, gp36: −)	HIV-1 positive
		476	Determine: non-reactive Uni-Gold: reactive ImmunoComb Combfirm: positive (p24+, p31−, gp120+, gp41+, gp36−)	Inconclusive	ELISA: reactive LIA: reactive (sgp120: − gp41: +/−, p31: −, p24: 2+ p17: 1+, sgp105: −, gp36: −)	Inconclusive (no DBS for PCR)

Determine: Determine HIV-1/2 (Alere, USA); ImmunoFlow: ImmunoFlow HIV 1–HIV 2 (Core Diagnostics, UK); Uni-Gold: Uni-Gold HIV (Trinity Biotech, Ireland); Stat-Pak: HIV 1/2 Stat-Pak (Chembio, USA); ImmunoComb CombFirm: ImmunoComb II HIV 1&2 CombFirm (Orgenics, Alere, Israel); EIA GS: GS HIV-1/HIV-2 PLUS O EIA (Bio-Rad, USA); BiSpot: ImmunoComb II HIV 1&2 BiSpot (Orgenics, Alere, Israel); DBS: dried blood spot.

Of the two clients in Kitgum classified as having acute infections, one was diagnosed as positive by the national algorithm on site (indeterminate by the MSF algorithm), and the other as negative.

## Discussion

Reports of unacceptably high error rates in RDT-based HIV testing in some resource-constrained settings [5–17] led us to conduct a large, multisite study to assess the performance of testing algorithms used at six sites in sub-Saharan Africa. The results of the HIV testing algorithms routinely used in these sites were compared to those of an internationally recognized reference algorithm from the AIDS reference laboratory for HIV at ITM, Antwerp. Here, we show that the performance of testing algorithms failed to meet the WHO-recommended thresholds of a PPV of ≥99% [1] at Kitgum, Arua, Douala and Baraka. However, at Kitgum and Baraka, the 99% threshold could be exceeded when using an alternative algorithm including a simple confirmatory assay.

While the sensitivity and NPVs of testing algorithms were excellent (100%) at three of the six study sites (Conakry, Doula and Baraka), results at other sites showed lower sensitivities and NPVs, particularly after adjustment for the under-representation of algorithm-negative specimens. Indeed, in sites such as Arua, where less than 10% of those screened negative on site were included in the study, finding one false-negative study participant might mean that up to 10 false-negative clients would have been found if all had been tested using the reference standard. However, all false-negative results were later found to be positive when samples were tested a second time using the same on-site algorithm, except in Kitgum, where backup samples were not available at the end of the study. This finding suggests that most false-negative results could have been due to an improper procedure or misinterpretation of the test in Arua. Alternatively, this difference could be attributed to the specimen used; while initial testing was performed on capillary whole blood at these sites, the backup sample was plasma. Although manufacturers are requested to show equivalence between recommended sample types, RDTs on serum/plasma have been reported to have higher sensitivity and lower specificity compared to RDTs on capillary whole blood [22,23].

The on-site algorithms specificities and PPV were also suboptimal (i.e. PPV <99%) in four sites using national or local algorithms (Kitgum, Arua, Douala and Baraka). We identified clients who had been misdiagnosed as false-positives at all but one study site. Most of the false-positive results were confirmed upon retesting of the backup sample. In Douala, six of eight clients with an initial false-positive result remained false-positive with the RDT algorithm using a fresh sample but were found to be negative by the reference test, indicating an intrinsic problem with the RDT algorithm.

Several reasons could be proposed to explain the suboptimal PPV of these RDT algorithms including low specificity of individual RDTs. First of all, it should be noted that none of the algorithms used in the study sites followed the current WHO guidelines for HIV testing [1]. Although the use of a tiebreaker is not recommended by the WHO, it is common in the WHO African region [4] and a tiebreaker was used in three of our study sites. The use of a tiebreaker has been associated with a higher risk of false-positive results [1,9,24]. However, in this study, only one of 16 false-positive results was due to the use of a tiebreaker, whereas all others had reactive results with the first two assays. This suggests that the specificity issues encountered here were due to shared cross-reactivity between tests. With the exception of the ImmunoFlow and the ImunoComb II HIV 1&2 Combfirm, all tests are WHO prequalified and have shown good specificity in prequalification evaluations [25]. However, data from different studies suggest that their performance in real-life conditions may vary [5,7–17].

In response to previous reports of false-positive results, MSF proposed an alternative algorithm containing a simple confirmatory assay to reduce the number of false-positive results [7]. Using this assay in Baraka increased the specificity and PPV of the alternative algorithm, as compared to the local algorithm. However, the use of a simple confirmatory assay, as opposed to a RDT-only algorithm, still needs to be evaluated and balanced with cost and ease of use. The use of a third RDT to confirm a positive result, as proposed in the latest WHO recommendations for low-prevalence settings, might also increase the PPV of the algorithm [1].

As is common practice in diagnostic test evaluations, inconclusive results were excluded from the analysis of sensitivity and specificity. However, given their significant psychological and pragmatic impact, an overall performance evaluation of the algorithm should take these into consideration. Here, the proportion of inconclusive results among study participants should be interpreted with caution since it was not representative of the overall proportion due to our sampling strategy. It is also important to note that inconclusive results were only reported in sites where a tiebreaker was not used. In these sites, the overall proportion of inconclusive results varied from 0.2% to 1.9%. Whether this reflects geographical variability or is caused by immunological factors, as reported previously [26,27], or differences in testing algorithms should be investigated further.

The current WHO guidelines for HIV testing suggest performing a third RDT following discordant results with the first two RDTs, and, if this third RDT is non-reactive, recording the final result as negative. Importantly, this third RDT is not used as a tiebreaker since a reactive result with this third RDT would lead to an inconclusive result. Still, this approach would likely yield a smaller proportion of inconclusive results, most of which, as we have shown, were found to be negative by the reference standard.

There were several limitations to this study. First, due to the low prevalence of HIV in certain sites, we selected study participants based on their on-site test results introducing a verification bias. We attempted to correct for this bias using a method proposed by Zhou [21], but this led to wide confidence intervals for some estimates. Additionally, while several sites performed on-site testing on capillary whole blood, retesting of backup samples and reference test was always performed on plasma. Though this allows for a performance assessment of the algorithms in real-life conditions, it restricts investigation of false results. The lack of systematic testing of backup samples in certain sites also made it difficult to discern the cause of discrepancies between on-site and reference results.

### Conclusions

This large multicentre study of the performance of HIV testing algorithms in sub-Saharan Africa highlights the inconsistent performance of HIV testing algorithms. While suboptimal sensitivities of testing algorithms could be the product of procedural mistakes, an inadequate RDT algorithm in Douala was responsible for suboptimal specificity and PPV. Alongside quality issues, such as respecting incubation time, correct labelling, batch control and careful selection of HIV, RDTs for the algorithms in use should be conducted regularly in order to minimize the risk of misdiagnosis. National authorities should also ensure that their policy aligns with the most current WHO recommendations, in terms of both algorithm design and implementation of other strategies to mitigate against misdiagnosis, such as retesting at the start of antiretroviral therapy.
